# Extraction of Prostatic Lumina and Automated Recognition for Prostatic Calculus Image Using PCA-SVM

**DOI:** 10.1155/2011/831278

**Published:** 2011-02-24

**Authors:** Zhuocai Wang, Xiangmin Xu, Xiaojun Ding, Hui Xiao, Yusheng Huang, Jian Liu, Xiaofen Xing, Hua Wang, D. Joshua Liao

**Affiliations:** ^1^Department of Pathology, Liuhuaqiao Hospital, Guangzhou, Guangdong 510010, China; ^2^School of Electronic and Information Engineering, South China University of Technology, Guangzhou, Guangdong 510640, China; ^3^Institute of Electronics and Information, Guangdong Institute of Science and Technology, Guangzhou, Guangdong 510640, China; ^4^Hormel Institute, University of Minnesota, Austin, MN 55912, USA

## Abstract

Identification of prostatic calculi is an important basis for determining the tissue origin. Computation-assistant diagnosis of prostatic calculi may have promising potential but is currently still less studied. We studied the extraction of prostatic lumina and automated recognition for calculus images. Extraction of lumina from prostate histology images was based on local entropy and Otsu threshold recognition using PCA-SVM and based on the texture features of prostatic calculus. The SVM classifier showed an average time 0.1432 second, an average training accuracy of 100%, an average test accuracy of 93.12%, a sensitivity of 87.74%, and a specificity of 94.82%. We concluded that the algorithm, based on texture features and PCA-SVM, can recognize the concentric structure and visualized features easily. Therefore, this method is effective for the automated recognition of prostatic calculi.

## 1. Introduction

The prostate consists of the gland and the stroma. The prostate is a collection of 30 ~ 50 branched tubuloalveolar glands whose ducts empty into the prostatic urethra. The prostate produces prostatic fluid and stores it in its interior for expulsion during ejaculation. A fibroelastic capsule rich in smooth muscle surrounds the prostate. This capsule emits septa that penetrate the gland. An exceptionally rich fibromuscular stroma is formed that surrounds the gland. The basal lamina is indistinct, and the epithelial cells rest upon a layer of connective tissue with much smooth muscle, a dense elastic fiber network, and blood capillaries. Its epithelium may be cuboidal or even squamous but in most places is columnar, with a few basal cells, which secrete proteins. The prostatic gland is divided into 3 glandular components, that is, mucosal, submucosal, and main glands, arranged in 3 separate areas situated concentrically around the urethra. The main glands contribute most to the volume of the prostatic secretion. Small spherical bodies of glycoprotein composition less than 0.2 mm in diameter are frequently observed in the lumen of the prostate. They are called prostatic concretions, or corpora amylacea. These bodies often form calculi.

Identification of prostatic calculi is an important basis for determining the tissue origin. Prostate histology images appear to be different types. Even the same type still has different texture and appearance. A prostate calculus looks similar to other materials and often attaches to other tissues in histology images. In this communication, we proposed local entropy, Otsu threshold, and mathematical morphology methods to extract the prostatic lumina and to isolate out the suspicious calculus regions. Automated recognition of prostatic calculi, which is based on the texture features of the prostatic calculus and a PCA-SVM model, can simplify the algorithm and improve the recognition accuracy. 

## 2. Materials and Methods

### 2.1. Materials

The samples were all from Liuhuaqiao Hospital of Guangzhou, archived from January 2003 to December 2007, and consisted of benign prostatic hyperplasia, 13 cases, ages 61 to 79, averaging 65.7, whose course of disease was about 1 to 12 years, and prostatic cancer, 15 cases, ages 63 to 75, averaging 67.3, whose course of disease was about 3 to 6 years. The two groups of samples shared no significant differences in age and course of disease (*P* > .05). Among all these cases, specimens of 6 cases of benign lesions, and 11 cases of prostate cancer were achieved by biopsy, with 7 cases of benign lesions and 4 cases of prostate cancer achieved by radical retropubic prostatectomy. All specimens were prepared by 10% neutral formalin fixation, embedded in paraffin, sliced for pathology to 5 *μ*m thickness, stained by H&E and were double-blind evaluated by two senior pathologists.

### 2.2. Methods

#### 2.2.1. Images Acquisition

Focusing on the calculi in the lumina, images with different magnification of 10 and 20 times were obtained seamlessly by an Olympus BH4 Microscope and then were captured in about 30 images for analysis by a Canon digital camera. The light source of the system was adjusted by blank field of vision.

#### 2.2.2. Extraction of Lumina and Suspicious Calculus Regions

**Local Entrop**

*f*(*i*, *j*) is the gray of (*i*, *j*),*f*(*i*, *j*) > 0, for an image of *M* × *N*, we define that [1]

(1) $$H_{f}=-\overset{M}{\underset{i=1}{\sum }}\overset{N}{\underset{j=1}{\sum }}p_{ij}\log\;p_{ij},\;p_{ij}=\frac{f\left(i,j\right)}{\sum_{i=1}^{M}\sum_{j=1}^{N}f\left(i,j\right)},$$

where *H*_*f*_ is the entropy, and *p*_*ij*_ is the probability distribution of gray. If *M* × *N* is the local window, then *H*_*f*_ is the local entropy.

**Otsu Method**

Using the Otsu method by selecting a proper threshold value, the disparity between foreground and background would be obvious as possible. If the average gray of the foreground is *μ*_0_, whose probability is *ω*_0_, and the average gray of background is *μ*_1_, whose probability is *ω*_1_, then the average gray of the image is *μ* = *ω*_0_ *μ*_0_ + *ω*_1_ *μ*_1_. The best threshold value is *T* which makes *g* = *ω*_0_(*μ*_0_−*μ*)^2^ + *ω*_1_(*μ*_1_−*μ*)^2^ the max [2].

**Mathematical Morphology**

Mathematical morphology [3], which includes Expansion, corrosion, open operation and close operation, and regional filling methods, was used to extract the luminal regions completely and prevent needless adhesion between calculus and calculi and lumina. The Otsu method and mathematical morphology were used to segment the suspicious calculus for further evaluation.

**Calculus Recognition Based on PCA-SVM**

In the prostate histology images, the prostatic calculus is round or oval shaped with a structure of concentric lamellar texture features. From the characteristics of the calculus, establishing a PCA-SVM model to distinguish real calculus region from noncalculus regions would be possible.

**Texture Features**

Texture features of the gray image consisted of statistical, spectral, and structural characteristics. They were correlated to the location, shape, size, and direction of the object, but not to the average gray level. In this paper, we calculate the gray level co-occurrence Matrix of the suspicious calculus, and analyze the 70 texture features of each suspicious calculus

- *Gray Level Co-Occurrence Matrix*. Gray level co-occurrence matrix (GLCM) is a well-known method for analyzing texture images. We define GLCM as [*p*(*i*, *j*, *θ*, *d*)], where *i*, *j* = 0,1,…, *L* − 1 are the gray levels of the image. The GLCM values were computed for the four basic directions *θ* = 0,45,90,135° and for *d* = 1 pixel. We also computed the mean of the four basic directions as the fifth direction.
- *Texture Features*. Normalized the five GLCM: *p*(*i*, *j*) = *p*(*i*, *j*)/∑_*i*,*j*_ *p*(*i*, *j*).
- We used 14 types of texture features in our study, including energy, contrast, correlation, entropy, variance, sum of average, sum of variance, homogeneity, variance of difference, sum of entropy, difference of entropy, shadow of clustering, prominence of clustering, maximal probability. Namely, 70 features were acquired from each suspicious calculus region.

#### 2.2.3. Principal Component Analysis

Principal component analysis (PCA) allows us to compute a linear transformation when the mapped data is from a high-dimensional space to a lower-dimensional space. It is an unsupervised linear analysis method, which can reduce the impact on the classifier caused by correlated texture features. So, we extract the principal component, whose contribution rate is larger than 98% (*α* ≥ 98%), as the input of SVM classifier.

#### 2.2.4. SVM Classifier

The kernel SVM classifier converts a low-dimensional vector space map to a higher dimensional space and a nonlinear question to a linear one, and then chooses the best category surface to solve the classification problem. In this paper, we used the traditional SVM classifier method. The standardized data obtained through PCA is different from the original and cannot be used in a trained SVM classifier for classification. Therefore it is very necessary to deal with the features of test images and training set together to implement principal component analysis. As we perform PCA, we combine the train and test date before applying PCA. And then, the new combinatorial features are served as data for SVM classifier.

## 3. Results

### 3.1. Homogeneous Area Extraction Based on Local Entropy

**Figure 1:**
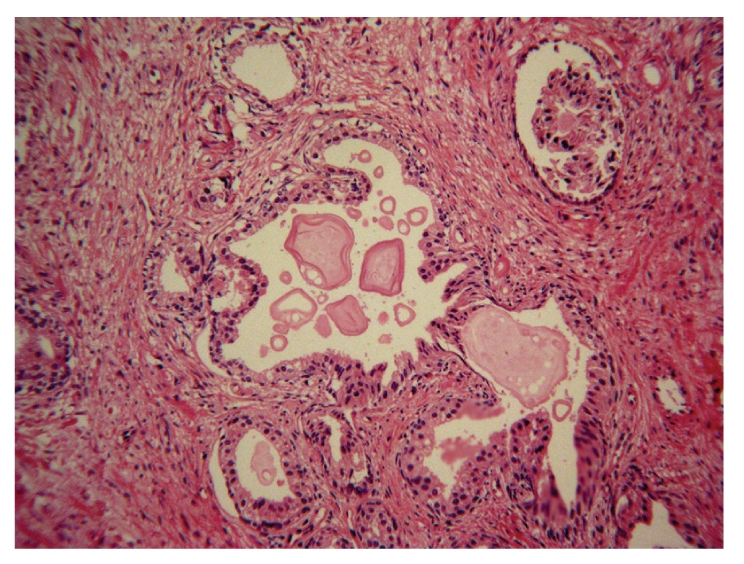
Fibromuscular stroma surrounds the prostatic lumina. Dilated prostatic lumina contain various prostatic calculi.

**Figure 2:**
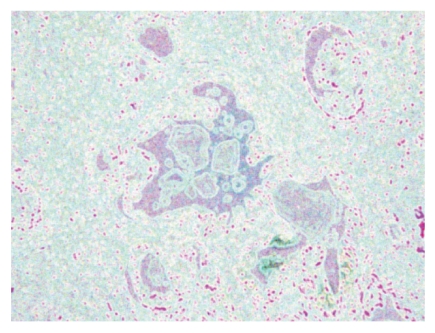
The prostate histology image reveals blue and red texture through the local entropy.

The local entropy is found by an image transformation method. First, a square neighborhood of each pixel is designated. Then, the entropy of the gray value of the pixels in this region is computed and then replaced by the gray value with the entropy. The smaller gray-scale changes, the smaller its entropy is. The cell nucleus of glandular epithelium and stroma and the variance of textures of the fibrous connective tissue are the factors that lead to the mutation of gray in the prostate histology images and the change in local entropy. But the textures of the lumina are relatively homogeneous, which makes it possible to extract the lumina based on the local entropy (Figures 1 and 2). Here, we first transformed a color image into three gray images, and then the local entropy method is applied in each image. Three transformed images are composed in Figure 2.

### 3.2. Conversion of the Gray Image into Binary Based on Threshold

**Figure 3:**
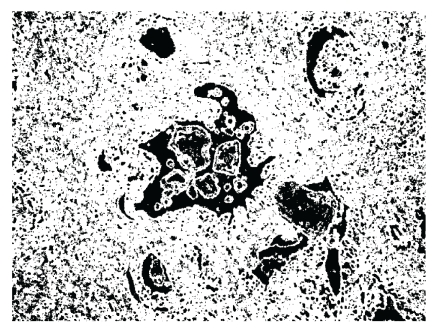
The lumina and the background are converted from a gray image to a binary image based on Otsu threshold.

Calculate the optimal threshold to separate the lumina and the background, resulting in a binary image (Figure 3).

### 3.3. Extraction of Lumina and Suspicious Calculus Based on Mathematical Morphology

**Figure 4:**
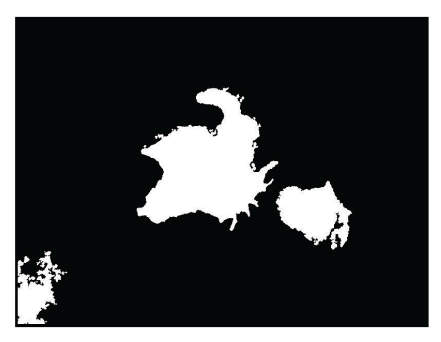
The prostate lumina are extracted completely through morphological processing.

**Figure 5:**
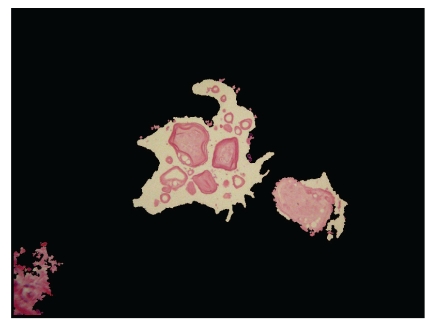
The extracted prostate lumina contain various calculi and adhesion.

**Figure 6:**
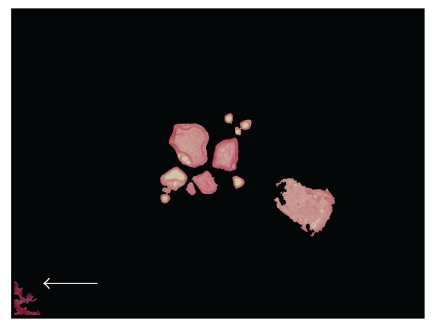
The suspicious calculi were segmented from the prostate lumina. The arrow points out the adhesion in the prostate lumina.

Mathematical morphology that included expansion, corrosion, open operation and close operation and regional filling methods were used to extract the lumina completely and to prevent needless adhesion between calculus and calculi and lumina (Figures 4 and 5). The Otsu method and mathematical morphology was used to segment the suspicious calculus for further evaluation (Figure 6).

### 3.4. Texture Features

Calculi are round or of oval structures with concentric lamellar texture features and without directional characteristics. By calculating the GLCM on the five directions to obtain 14 types of texture features, 70 features were gotten for each suspicious calculus region. The PCA method was introduced to extract the principal component, whose contribution rate was larger than 98% (*α* ≥ 98%), as the input of SVM classifier.

### 3.5. Integrated PCA on Multivariate Map Data

Deal with the 70 features of each suspicious calculus: (1) standardize the matrix of the data sets, (2) calculate the correlation coefficient matrix of variables, (3) calculate the matrix eigenvalue of the variable, (4) determine the number of principal components (*α* ≥ 98%), and (5) calculate the value of each principal component. The segmentation of the prostate histology image resulted in 172 suspicious calculus regions. Among them, the chosen samples were 28 real calculus regions and 99 noncalculous regions which were unconnected with the edge of image. Restricting contribution rate to above 98%, we got 10 components by calculating 70 texture features of corresponding samples based on PCA. Here, we only get 10 combined components not the 70 original features, so the contribution rate of 10 components was above 98%, not 100%. Of the 127 samples, two are unsatisfactory and thus culled away in order to apply a 5-fold cross validation during the following SVM classifier.

### 3.6. Recognition of Calculus by PCA and Data Mining

**Figure 7:**
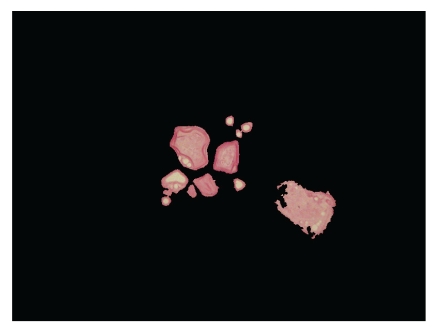
The image of automated recognition of prostate calculi appears as light blue or red white concentric circle structures of tree-ring like texture. The prostate luminal adhesion in the left- bottom corner disappears.

After using PCA method on the 70 texture features, a 5-fold cross validation was applied to training and testing samples. The SVM was used to recognize calculi (Figure 7). The kernel of the SVM classifier was radial basis function (RBF). One time of 5-fold cross validation in our experiments can be divided into the following steps: (1) 125 samples are initially divided into 5 sets and each set has 25 samples, (2) at the first step, the first, second, third, and fourth sets served as training samples, and the fifth set served as the test samples, (3) at the second step, the first, second, third, and fifth sets served as training samples and the fourth set served as the test samples, (4) do the similar step, until at the fifth step, the second, third, fourth, and fifth sets served as training samples, and the first set served as the test samples.

In such a process, every sample served as both training sample and test sample. It is susceptible and nonaccurate to evaluate the performance of a new method based on a few samples such as one step of one time of 5-fold cross validation. Therefore, we analyze statistically after one time of 5-fold cross validation, which includes the five steps described above. 

In a cross-validation process, 125 samples have been tested in turn, then the statistics corresponding true negative samples (TNSs), false positive samples (FPSs), false negative samples (FNSs) and true positive samples (TPSs) values can be known, so we can calculate the accuracy, specificity, sensitivity, and so forth. We have done the 5-fold cross validation for ten times based on the same samples but different combinations of sets, as at the beginning of each time of validation, the samples are divided randomly. The results of ten times of 5-fold cross validation are shows in Table 1. Each column in Table 1 represents the result of one time of 5-fold cross validation. 

The results in Table 1 showed that the average training accuracy was 100%, the average test accuracy was 93.12%, sensitivity was 87.74%, and specificity was 94.82%, respectively. The algorithm of the recognition for prostate calculus we presented in this paper achieved good results with a high rate of accuracy, sensitivity and specificity. And the specificity was much higher than the sensitivity. All the above results suggested that the possibility of misdiagnosis of calculus would be low.

## 4. Discussion

Prostatic calculi are the outcome of the degeneration of prostate tissues, which have a high incidence with increasing age. Calculi locate in the lumina, are small round or oval shape with a smooth surface and a hard structure, and appear to be light blue or red with white concentric circles structure of tree-ring-like texture under the microscope. These unique characteristics of color, texture, and appearance are the main bases for the identification of prostatic tissue as the source of a specimen. 

At present, research on computer-aided diagnosis for prostate histology images focuses on how to make pathological grade accurate and to have high robustness. On the classification studies, Smith [4] 4-class and Farjam [5] 5-class were comparable to Gleason grade, but the accuracy of the latter was still only 85%. For the pathological features studies, the classification on prostate cancer G2–G5 was acquired based on texture features [6], or combination of morphological and texture features [7]. There were also some researches based on a same classifier shared difference on the algorithm [8], quantitative analysis [9], and morphology [10]. We presented the result of the automatic classification based on the combination study of morphological and texture features [11]. 

The images were computer analyzed using methods including extraction of the lumina and suspicious calculi based on local entropy, Otsu thresholding and mathematical morphology, calculation of the gray level co-occurrence Matrix of the suspicious calculi and 70 texture features of each suspicious calculus, the analysis of texture features by PCA to reduce the effect on the SVM classifier, choosing the component whose contribution was above 98% as the input of the classifier, and the result demonstrating an accuracy rate of 93.12% for the recognition of single calculi. The tissue structure and cell morphology of various areas were well represented by prostate histology images, which were obtained seamlessly by a microscope. Images acquired with different magnifications at the same area would also be obtained for detail observation. Pathological diagnosis made was based on the integrated different analysis of tissue structure and cell morphology on many images. With multiple magnifications, and with many images containing one or more calculus, there was little tendency for misdiagnosis by the computer, with a calculus recognition rate of 93.12%. It was felt that the computer diagnosis would be less influenced by human factors and would have a lower possibility of misdiagnosis. 

In conclusion, based on texture features and PCA-SVM, our algorithms that extract the lumina, segment the suspicious calculus, and recognize calculi in an automated fashion made it possible and easy to recognize the concentric structure and the visualized features, reduce the complexity of the algorithm and improve the accuracy of diagnosis.

## Figures and Tables

**Table 1:** Each column shows results of one time of 5-fold cross validation, which includes the five steps described.

	1	2	3	4	5	6	7	8	9	10
ATT (s)	0.1438	0.144	0.1532	0.1436	0.1314	0.1312	0.1374	0.1594	0.153	0.1346
AART (%)	100	100	100	100	100	100	100	100	100	100
ATA (%)	92.8	92	93.6	92.8	94.4	93.6	94.4	91.2	94.4	92
TNS	91	90	93	93	93	92	94	92	95	90
FPS	6	7	4	5	5	5	4	5	2	7
FNS	3	3	4	4	2	3	3	6	5	3
TPS	25	25	24	23	25	25	24	22	23	25
Sensitivity (%)	89.5	89.3	86.7	83.8	92.5	89.8	91.3	78.6	84.5	91.4
Specificity (%)	94	92.8	95.9	94.7	94.9	94.8	96.3	94.7	97.8	92.3

ATT: average training time; AART: average accuracy rate of training; ATA: average test accuracy; TNS: true negative samples; FPS: false positive samples; FNS: false negative samples; TPS: true positive samples.
